# Frontal Balloon Sinuplasty in Complicated Acute Pediatric Rhinosinusitis (ARS)

**DOI:** 10.1155/2022/7232588

**Published:** 2022-05-14

**Authors:** Smrithi Chidambaram, Benjamin M. Wahle, David S. Leonard

**Affiliations:** ^1^Department of Otolaryngology – Head and Neck Surgery, Washington University in Saint. Louis, School of Medicine, St Louis, MO 63110, USA; ^2^Saint Louis University School of Medicine, St. Louis, MO 63104, USA

## Abstract

Utilization of frontal balloon sinuplasty in pediatric complicated acute rhinosinusitis (ARS) is demonstrated to be a safe and expedient alternative to other procedures such as trephination or functional endoscopic sinus surgery (FESS) in this case series. We performed a retrospective review of six pediatric cases of frontal balloon sinuplasty for ARS with intracranial complications at a tertiary academic center. Patients underwent unilateral (*n* = 5) or bilateral dilation (*n* = 1) in addition to functional endoscopic sinus surgery (FESS) including anterior ethmoidectomy (*n* = 5) and maxillary antrostomy (*n* = 6). This technique effectively addressed frontal sinus obstruction and served as an alternative to procedures such as trephination or functional endoscopic sinus surgery. No immediate or short-term complications of balloon dilation were observed in these cases. A larger cohort and extended follow-up are necessary to determine the use and long-term impact of this technique.

## 1. Introduction

Acute rhinosinusitis (ARS) is a significant disease among pediatric patients and a common complaint in the primary care setting [1]. Of pediatric patients with upper respiratory infections, 5–13% progress to a diagnosis of ARS [2]. Although rare, intracranial complications of ARS including cavernous sinus thrombosis, epidural abscess, cerebral abscess, meningitis, subdural empyema, and cerebritis can be life-threatening and require prompt intervention [3–6]. Recent studies suggest that orbital or intracranial complications occur in less than 1% of patients diagnosed with ARS in the emergency department, while 4.3% of pediatric ARS inpatient admissions involved intracranial complications [7, 8]. In one institutional cohort study, 30.4% of pediatric frontal sinusitis cases had intracranial complications [9]. This specific paranasal sinus is more commonly associated with intracranial complications and worse outcomes, including need for surgical intervention and prolonged hospital stay [9, 10].

It is established that frontal sinus involvement is common in ARS with intracranial complications, and it represents a surgical challenge [9, 11]. Traditional surgical approaches for frontal drainage include functional endoscopic sinus surgery (FESS) and trephination. Frontal FESS can be technically challenging in the setting of ARS, and otolaryngologists may vary in level of experience with pediatric frontal FESS during ARS. Trephination provides a more expedient approach to frontal drainage but carries a risk of cosmetically significant scarring. As balloon dilation is becoming more commonly utilized in the United States population for frontal sinus disease, more attention is being placed on the utilization of and outcomes following this procedure and in specific subpopulations [12].

Treating complicated pediatric frontal ARS with balloon sinuplasty has been previously described in one case report of a patient with an intracranial abscess and in a case series of four patients with the following complications: forehead swelling, frontal dural enhancement, intraorbital manifestation, and recurrent chronic sinusitis [13, 14]. Multiple studies have established that balloon sinuplasty is safe and useful when examining short and long-term outcomes in pediatric chronic rhinosinusitis (CRS), though a consensus has not been reached for the indication of balloon dilation among children with CRS [15–23]. A prospective study which included 30 pediatric frontal dilations demonstrated this technique to improve quality of life in pediatric CRS [16]. Balloon sinuplasty has also been found to be effective in adults with recurrent frontal ARS [24]. As research on the safety and efficacy of frontal balloon dilation in the context of pediatric ARS with intracranial complications is limited, this subject merits further examination.

## 2. Case Presentation

A retrospective chart review of six pediatric cases of frontal balloon sinuplasty for complicated ARS was performed with approval from the Human Research Protection Office at Washington University in St. Louis. Eligible patients were less than 18 years of age and had an operating room charge for a sinus balloon between January 2017 and June 2019. Patients with CRS and/or balloon dilation of the maxillary sinus only were excluded.

Frontal balloon dilation utilizes the same equipment, positioning, and navigation systems as FESS. After nasal mucosal decongestion and injection, limited FESS is performed. This includes middle turbinate medialization, uncinectomy, maxillary antrostomy, and anterior ethmoidectomy. The frontal recess is identified in all patients using image guidance, and a guidewire with a lighted tip is inserted. Transillumination of the frontal sinus visible through the forehead confirms successful guidewire placement. A sinus balloon is then inserted over the guidewire into the frontal recess and inflated per the manufacturer's specifications. The balloon dilates the sinus ostium, and the sinus is irrigated until drainage returns clear.

### 2.1. Preoperative Patient Characteristics

The characteristics of the study population are given in Table 1. The median age of patients was nine (range 7–14) with an even distribution of males and females. The significant medical history included three (50%) cases of allergic rhinitis and one case of a recent frontal bone fracture. All patients were initially imaged with CT and MRI. All cases involved the frontal, maxillary, and ethmoid sinuses; only one case had sphenoid sinus involvement. All cases included intracranial complications with either subdural empyema (50%) or epidural abscess (50%). Other complications included frontal cerebritis, frontal soft tissue abscess, and frontal osteomyelitis. Three (50%) patients were neurologically intact upon admission, while two patients reported symptoms concerning for seizure and one patient demonstrated altered mental status (GCS 12).

### 2.2. Management and Postoperative Course

**Figure 1:**
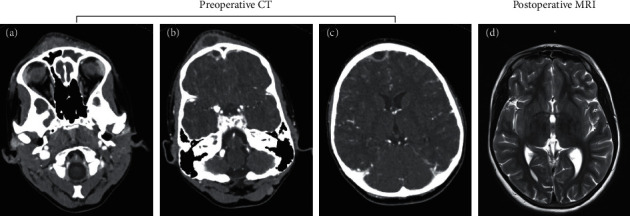
Imaging findings before and after balloon dilation. (a)–(c) Preoperative head CT slices with contrast of one case included in the study: (a) fluid-density opacification of the frontal sinus; (b) contrast-enhancing subperiosteal fluid collection overlying the frontal bone, consistent with Pott's puffy tumor; (b)-(c) contrast-enhancing intracranial fluid collection consistent with epidural abscess. (d) T2 MRI six weeks after surgery revealing resolution of abscesses and aeration of the frontal sinus.

Surgical management and follow-up are also given in Table 1. While all cases were treated with frontal balloon sinuplasty, bilateral dilation was only performed on one patient. Postoperatively, patients were managed with oxymetazoline, nasal saline irrigations, and topical corticosteroid sprays. Neurosurgical procedures were required in four (67%) patients. All patients were treated with 4–8 weeks of IV antibiotics. Hospital admission periods ranged from 4 to 43 days (median 13.5 days). Median follow-up time was 91 days. Three (50%) patients had hospital readmissions following their ARS surgeries. One patient had three readmissions for unrelated diagnoses. Two others were admitted for fever workup given their recent hospitalizations. No diagnoses of ARS or intracranial infection were made during these readmissions.  Figure 1 shows representative imaging before and after balloon dilation of one case included in the study. Figures 1(a)–1(c) show three frames from a preoperative head CT that highlights multiple potential complications of pediatric ARS patients with intracranial complications including fluid-density opacification of the frontal sinus (Figure 1(a)), subperiosteal fluid collection overlying the frontal bone consistent with Pott's puffy tumor (Figure 1(b)), and epidural abscess (Figures 1(b) and 1(c)). In this case, MRI six weeks after surgery demonstrates resolution of the abscesses and aeration of the frontal sinus (Figure 1(d)).

## 3. Discussion

In our practice, frontal balloon sinuplasty in pediatric ARS with intracranial complications allows for relatively expedient source control with limited instrumentation of the frontal recess. While this may be achieved with traditional frontal FESS techniques, this is technically challenging in setting of inflamed, bleeding mucosa and may increase operative time and risk. Unlike trephination, frontal balloon sinuplasty can address anatomic obstruction of the frontal sinus without any external scarring. In 2011, 11.8% of pediatric frontal sinus procedures utilized balloon catheter dilation in the United States; while balloon sinuplasty carries additional equipment cost when compared to traditional FESS alone, it is still unclear whether this cost might be mitigated by decreased surgical time using this technique [25, 26].

Similar to a pediatric case reported in 2016, no short-term complications of balloon dilation were observed in these six patients [13]. Though half of our patients had a hospital readmission, none were diagnosed with sinusitis or intracranial infections. Half the patients in this series presented without neurologic symptoms. Neurological deficit from epidural abscess is often delayed due to its insidious spread, and intracerebral abscesses can present without neurological symptoms [27]. Moreover, previous studies have shown that 63–72% of pediatric sinusitis patients with intracranial empyema or abscess require at least one neurosurgical intervention during their treatment similar to our cohort in which 67% of the cases required a neurosurgical procedure [3, 27]. Another case series examining the efficacy of frontal balloon sinuplasty in four pediatric patients with complicated acute frontal sinusitis demonstrated sustained symptom resolution 1–3 years following the procedure in three of the cases [14]. Although a larger patient cohort and extended follow-up are needed to establish the overall safety of frontal balloon sinuplasty in this population, our data adds to the literature as it demonstrates that frontal balloon dilation does not influence the need for additional neurosurgical procedures and is both safe and effective in treating complicated pediatric ARS.

## 4. Conclusion

In this case series, frontal balloon dilation is determined to be a safe and expedient approach in complicated pediatric ARS. Frontal balloon sinuplasty avoids the external scarring of trephination and the technical challenges associated with frontal FESS. While this study demonstrates the potential for utilizing frontal balloon sinuplasty in the context of complicated ARS in children, it is limited to a single institution and a small patient sample. A larger cohort and extended follow-up are required to determine the long-term efficacy and safety of this technique compared to other surgical modalities.

## Figures and Tables

**Table 1:** Demographic, clinical, and management characteristics of complicated pediatric ARS patients (*n* = 6).

	Median *n*(%)
Age	
Median (range)	9 (7–14)

Gender	
Male	3 (50)
Female	3 (50)
Medical history	
Allergic rhinitis	3 (50)
Recent frontal bone fracture	1 (17)
Initial imaging	
CT + MRI	6 (100)
Sinuses	
Frontal	6 (100)
Maxillary	6 (100)
Ethmoids	6 (100)
Sphenoid	1 (17)
Laterality	
Unilateral	1 (17)
Bilateral	5 (83)
Sinusitis complications	
Intracranial complications	6 (100)
Subdural empyema	3 (50)
Epidural abscess	3 (50)
Extracranial complications	3 (50)
Frontal cerebritis	1 (17)
Frontal soft tissue abscess	1 (17)
Frontal osteomyelitis	1 (17)
Neurologic presentation	
Intact	3 (50)
Concern for seizure	2 (33)
AMS (GCS 12)	1 (17)
Visits to operating room	
Number of ENT operations	
1	6 (100)
Number of neurosurgical operations	
0	2 (33)
1	3 (50)
2	1 (17)
Frontal sinus balloon dilation laterality	
Unilateral	5 (83)
Bilateral	1 (17)
Additional FESS performed	
Maxillary antrostomy	6 (100)
Anterior ethmoidectomy	5 (83)
Neurosurgical procedures	
Craniotomy and washout	3 (50)
Burr holes and washout	1 (17)
ICU admission	
Yes	4 (67)
No	2 (33)
Hospital readmission	
Yes	3 (50)
No	3 (50)
	Median (range)
Hospital duration	13.5 (4–43)
Follow-up time (days)	91 (38–406)

## Data Availability

The data used to support the findings of this study are available from the corresponding author upon request.

## Abbreviations

ARS: Acute rhinosinusitis
CT: Computerized tomography
MRI: Magnetic resonance imaging
AMS: Altered mental status
GCS: Glasgow Coma Scale
ICU: Intensive care unit
FESS: Functional endoscopic sinus surgery.
